# Overexpression of GINS4 Is Associated With Tumor Progression and Poor Survival in Hepatocellular Carcinoma

**DOI:** 10.3389/fonc.2021.654185

**Published:** 2021-03-25

**Authors:** Ziying Zhang, Peng Chen, Hui Xie, Peiguo Cao

**Affiliations:** ^1^ Department of Oncology, Third Xiangya Hospital, Central South University, Changsha, China; ^2^ Department of Urology, Xiangya Hospital, Central South University, Changsha, China; ^3^ Department of Thoracic Surgery, Second Xiangya Hospital, Central South University, Changsha, China

**Keywords:** hepatocellular carcinoma, GINS4, expression, prognosis, biological significance

## Abstract

**Purpose:**

Our research was aimed to identify the expression, clinical value and biological significance of GINS complex subunit 4 (GINS4) in hepatocellular carcinoma (HCC).

**Materials and Methods:**

GINS4 was initially screened through weighted gene co-expression network analysis (WGCNA). The TCGA, GEO, and TIMER databases were applied for analyzing the GINS4 mRNA expression in HCC. GINS4 protein levels were detected *via* immunohistochemistry (IHC). Receiver operating characteristic (ROC) curve was applied for estimating the diagnostic significance of GINS4 in HCC. Kaplan-Meier plots, Cox model, and nomogram were used to assess the prognostic performance of GINS4 in HCC. Nomogram validation was conducted through time-dependent ROC and decision curve analysis (DCA). The Wanderer, UALCAN, and DiseaseMeth databases were utilized to identify GINS4 methylation levels in HCC. Genes co-expressed with GINS4 in HCC were estimated through the TCGA, cBioPortal, and GEPIA. GO, KEGG, and GSEA unraveled the possible biological mechanisms of GINS4 in HCC.

**Results:**

WGCNA confirmed that GINS4 was one of hub genes significantly associated with histological grade of HCC. Multiple databases confirmed the significant upregulation of GINS4 in HCC tissues compared with non-tumor controls. IHC analysis of 35 HCC patients demonstrated that overexpressed GINS4 positively correlated with advanced TNM stage and poor pathological differentiation. GINS4 could effectively differentiate HCC cases from healthy individuals, with an AUC of 0.865. Increased GINS4 expression predicted unsatisfactory prognosis in HCC patients, especially in age >60 years, histological grade 1, HBV infection-negative, and occurring relapse subgroup. Nomogram incorporating GINS4 level and TNM stage displayed satisfactory predictive accuracy and clinical utility in predicting HCC prognosis. Upregulated GINS4 exhibited hypomethylated levels in HCC. Functional analysis indicated that GINS4 potentially positively modulated cell cycle and PI3K/AKT/mTOR pathway.

**Conclusion:**

GINS4 is overexpressed in HCC and is correlated with undesirable survival of HCC patients.

## Introduction

Liver cancer is the second primary reason for tumor-associated deaths globally, with approximately 841,000 new diagnoses and 782,000 deaths annually (1). Liver cancer kills approximately 383,000 people per year in China, occupying about 51% of liver cancer-related deaths globally (2). HCC, the primary subtype of liver cancer, accounts for 75–85% cases (3, 4). HCC can be triggered by multifarious risk factors, such as chronic hepatitis B virus (HBV) and hepatitis C virus (HCV) infections, aflatoxin exposure, alcoholic abuse, autoimmune hepatitis, and metabolic disorders (3, 5–8). Despite the rapid progression of therapeutic interventions (such as radiofrequency ablation, hepatic resection, hepatic transplantation, transarterial chemoembolization, and stereotactic body radiation), the prognosis of HCC patients is undesirable due to the occurrence of distant metastasis and the increased recurrence (9). Furthermore, the majority of cases are initially diagnosed at advanced HCC owing to the non-specific symptoms in early stage and the deficiency of sensitive diagnostic biomarkers, with a 5-year survival rate of lower than 20% (4, 10). Hence, deep comprehension of the underlying mechanisms concerning HCC progression is required to unravel novel diagnostic and prognostic molecular biomarkers and to develop new effective therapeutic strategies of HCC.

The GINS complex, a heterotetrameric structure composed of four different subunits (Sld5, Psf1, Psf2, and Psf3 from the Japanese go-ichi-ni-san representing 5-1-2-3, also known as GINS4, GINS1, GINS2, and GINS3 in human genome, respectively), can interact with Cdc45 and Mcm2-7 to form the eukaryotic replicative helicase CMG (Cdc45-Mcm helicase-GINS) complex that unties double-stranded DNA prior to moving the replication fork during chromosome duplication (11–13). The GINS complex, without prominent enzymatic activity itself, is pivotal to initiate and elongate chromosome replication through binding to and strengthening the enzymatic function of Mcm helicase (14, 15). GINS4, also known as SLD5, a vital component of GINS complex, exerts a momentous effect on the initiation and prolongation of DNA replication in the G1/S phase cell cycle in eukaryotes (16). GINS4 participates in modulating early embryogenesis in mice and maintaining cell cycle progression and genomic stability in Drosophila (17, 18), indicating its effect on tumorigenesis. Prior studies have demonstrated overexpression of GINS4 in multifarious human cancers tissues and tumor cell lines, including colorectal cancer (CRC) (19, 20), bladder cancer (21), non-small cell lung cancer (NSCLC) (22), gastric cancer (23), and pancreatic cancer (24). Greater expression level of GINS4 in above human tumors is positively correlated with malignant biological properties, such as tumor proliferation, colony forming ability, migration, and invasion as well as epithelial-mesenchymal transition both *in vitro* and *in vivo* (19–23). Additionally, survival analysis has revealed that patients with tumor (such as NSCLC, gastric cancer, CRC, and pancreatic cancer) characterized by high GINS4 expression have significantly diminished overall survival (OS) and disease-free survival (DFS) compared with those with lower GINS4 expression (19, 20, 22–24). Thus, GINS4 exerts a vital effect on the malignant progression of tumors and potentially serves as a valuable target for cancer therapy and diagnosis. Nevertheless, no report exists on the role of the GINS4 in HCC so far.

In our report, we investigated the expression, clinical significance, and potential biological functions of GINS4 in HCC based on multiple databases and experiment validation. Initially, the mRNA expression profiles and corresponding clinical information of 371 HCC patients from The Cancer Genome Atlas (TCGA) database and 713 HCC cases from multiple Gene Expression Omnibus (GEO) datasets were analyzed to compare GINS4 mRNA levels between HCC samples and adjacent liver tissues. Meanwhile, GINS4 protein expression was detected through IHC analysis of 35 clinical HCC samples and paired adjacent liver tissues. Secondly, ROC curve evaluated the diagnostic performance of GINS4 and AFP for HCC. The Kaplan-Meier curve, Cox regression models, nomogram, time-dependent ROC curve, and DCA investigated the prognostic performance of GINS4 in HCC. Finally, GINS4 methylation level and its association with clinicopathological factors of HCC were determined *via* the Wanderer, UALCAN, and human disease methylation (DiseaseMeth) database. Genes co-expressed with GINS4 were identified through TCGA, cBioPortal, and Gene Expression Profiling Interactive Analysis (GEPIA) databases. Multiple bioinformatics analysis methods, including Gene Ontology (GO), Kyoto Encyclopedia of Genes and Genomes (KEGG), and Gene Set Enrichment Analysis (GSEA) as well as Pearson correlation analysis, were used to predict the potential mechanism of GINS4 in HCC.

## Materials and Methods

### Collection of Clinical Specimens

**Figure 1:**
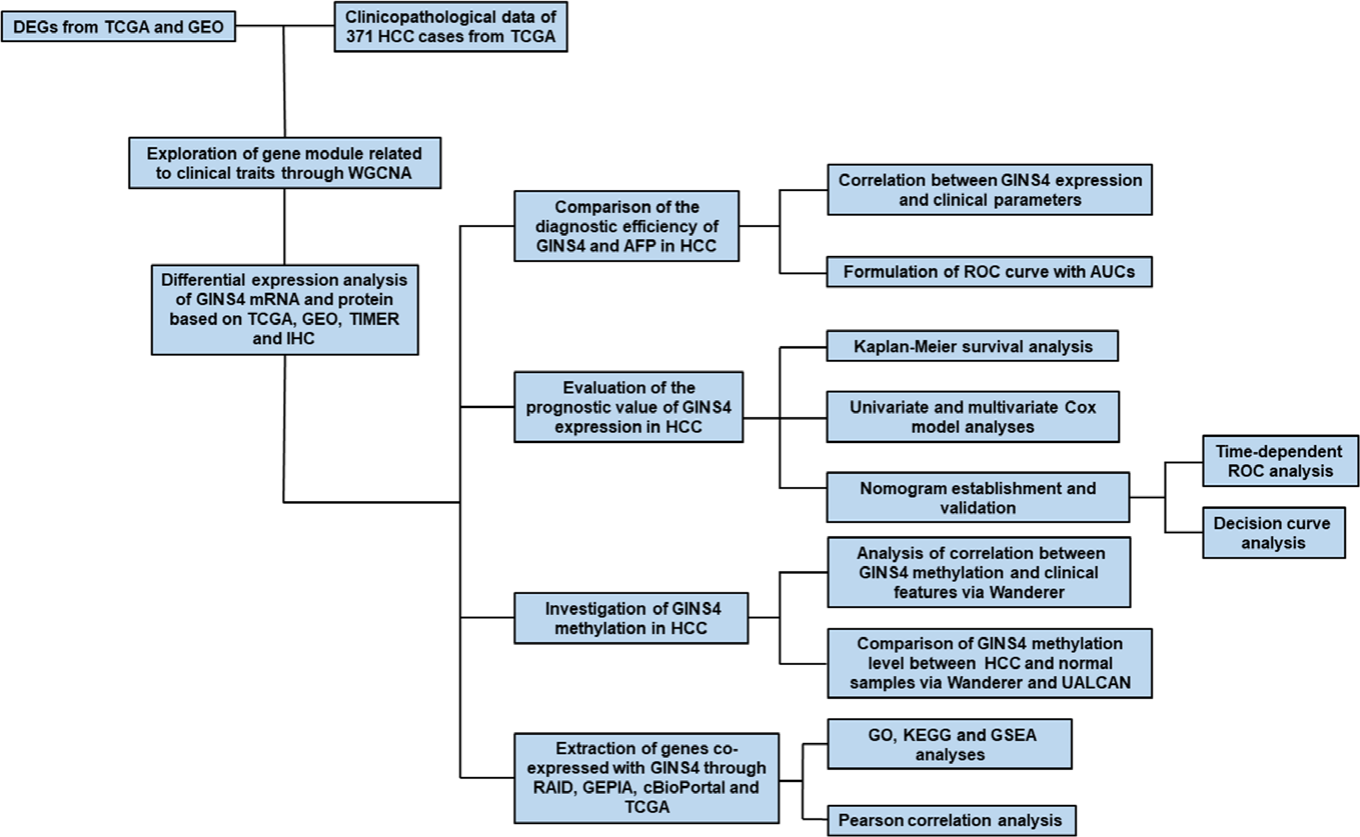
Flow chart of the present study. DEGs, differently expressed genes; WGCNA, weighted gene co-expression network analysis; IHC, immunohistochemistry; TIMER, the Tumor Immune Estimation Resource database; AUC, the area under the curve; ROC, the receiver operating characteristic; DCA, Decision curve analysis; GEPIA, Gene Expression Profiling Interactive Analysis.

The flowchart of our research was illustrated in **Figure 1**. From December 2017 and March 2020, a total of 35 paired surgically resected HCC samples and adjacent normal liver specimens were acquired from our hospital, which were used for IHC analysis. All primary HCC individuals had not accepted radiotherapy or chemotherapy before surgery. The present project was approved by the Ethical Committee for Clinical Research of our hospital. All volunteers conferred written informed consent to enroll in this project following receiving a full explanation of the purpose of the project.

### Public Database-Based Excavation of Data

The mRNA expression profiles extracted from the TCGA database (https://tcga-data.nci.nih.gov/tcga/) were normalized through “DEseq2” package (25). False discovery rate (FDR) <0.05 and |log_2_ fold change (FC) | > 1.0 were as thresholds for screening differently expressed genes (DEGs) between HCC tissues and adjacent normal liver samples. The heatmap and volcano map of DEGs were constructed by “pheatmap” and “ggplot2” R packages, respectively (5). The corresponding clinicopathologic variables was acquired from the TCGA GDC Data Portal database, including age at diagnosis, gender, ethnicity, body mass index (BMI), AFP level, fibrosis score, Child-Pugh classification, T classification, N classification, M classification, TNM stage, histological grade, tumor status, survival time, and survival status as well as whether had family history, viral hepatitis, hepatic inflammation, residual tumor, vascular invasion, relapse or not. We also used the “limma” package to investigate mRNA expression of DEGs between HCC samples and adjacent noncancer tissues in GEO dataset (http://www.ncbi.nlm.nih.gov/geo/), including GSE14520 (26), GSE25097 (27), GSE54236 (28), and GSE76427 (29).

### WGCNA Used for the Screening of GINS4

WGCNA is a novel systematic biology to unravel the association between gene networks modules and clinical phenotype at transcriptome level (30, 31). The dynamic tree cut approach was used to identify module. The modules with high similarity were estimated through cluster analysis. Modules could be merged when their correlation of module eigengene (ME) was higher than 0.95, indicating similar expression profiles among them. Pearson’s correlation analysis assessed the association between MEs and clinicopathological variables, such as gender, AFP level, Child-Pugh classification, TNM stage, histological grade, relapse, and survival status. Generally, the module with the largest absolute of module significance (MS) was selected for subsequent analysis. The gene significance (GS) and module membership (MM) were used to quantify the associations between each gene and external clinical traits in this module (31). Specifically, MM > 0.8 and GS > 0.2 were defined as the thresholds to screen hub genes in above target module. In our report, we utilized the “WGCNA” R package to formulate a co-expression network of 4344 DEGs in 371 HCC patients with corresponding clinical information (32).

### Immunohistochemistry

Resected specimens were fixed in 10% formalin, dehydrated, and embedded in paraffin. The paraffin specimens were segmented into 3 μm-thick sections and installed on the glass slide. Initially, the slides were incubated at 60°C for 30 min in a calorstat. Deparaffinization was carried out in xylene and rehydration was then performed in the gradient ethanol. Afterwards, the glass slides were boiled in EDTA solution (pH 8.0) for 5 min to block the endogenous peroxidase activity. Then, the sections were washed with PBS for three times and were then incubated with primary anti-GINS4 antibody (1:100; ab101346, Abcam, UK) at 4°C overnight. A secondary goat anti-rabbit antibody (1:200; ab205718, Abcam, UK) further incubated the slides at 37°C for 30 min. Binding of the primary antibodies was visualized *via* incubating chromogen diaminobenzidine (DAB, Sigma, UK) for 10 min at 37°C. The sections were counterstained with hematoxylin, dehydrated by a gradient ethanol, followed by xylene, and mounted (20, 23, 33).

The staining of each specimen was evaluated through two independent investigators blinded to the clinicopathological information. The GINS4 expression was considered positive when it was present in the membrane, the cytoplasm, or both. Each specimen was assessed at 200 and 400 magnification. The staining score was assessed according to two parameters: intensity and extension. The percentage of positively stained cells corresponded to five scoring grades: 0, less than 10%; 1, 10 to 25%; 2, 26 to 50%; 3, 51 to 75%; and 4, 76 to 100%. The intensity score was classified as 0, without staining; 1, yellow; 2, yellow-brown; 3, dark brown. The product of intensity and extension was identified as the total staining score which were stratified into three grades: 0 to 3, negative expression; 4 to 6, weakly positive expression; and more than 6, strongly positive expression (20, 23).

### Survival Analysis and Establishment of Nomogram

Based on the median GINS4 expression level, a total of 371 HCC cases were stratified into two groups (high *versus* low expression) in the TCGA database, respectively. The Kaplan-Meier survival curve with Wilcoxon rank sum test was formulated *via* “survival” R package to evaluate the OS and survival difference between high and low GINS4 expression groups (34). Univariate and multivariate Cox model was formulated for estimating the hazard ratio (HR) with 95% confidence interval (CI). The statistically significant prognostic factors identified by the univariate analysis were further incorporated into the multivariate analysis.

Nomogram is well-acknowledged model to predict long-term prognosis of patients with tumor (35, 36). The “rms” R package was applied for building a prognostic nomogram, thus estimating the probability of the 1-, 3-, and 5-year OS for HCC patients. Discrimination and calibration were used to validate the nomogram. The discrimination of the nomogram was estimated utilizing the concordance index (C-index) *via* a bootstrap method with 340 resamples. The calibration plot was applied for assessing the consistence between nomogram prediction and practical observation.

### Receiver Operating Characteristic Curve and Decision Curve Analysis

The time-dependent ROC curve and its corresponding area under the curve (AUC) value were established through “survivalROC” R package, thus assessing the discriminative accuracy of the predictive nomogram. The AUC value ranges from 0 to 1. The model presents a perfect discrimination when the AUC value is equal to 1. Conversely, the AUC value of 0.5 indicates a random capability to discriminate outcome (37).

DCA is a novel approach to assess the potential clinical net benefit (NB) of prognostic prediction models and to formulate better clinical strategies (38). NB is defined as a pivotal value that sums the benefits (true positives) and subtracts the harms (false positives) (37, 39). It can be plotted for a range of reasonable exchange rates in a decision curve where the potential utility of each decision strategy at each threshold probability is visualized (40). In the present study, we developed DCA by “rmda” R package, thus comparing the clinical utility of nomogram model, TNM stage, and GINS4 expression level (41).

### Analysis of GINS4 Methylation in HCC

To explore the mechanism of the dysregulation of GINS4 in HCC, the DiseaseMeth database (http://biobigdata.hrbmu.edu.cn/diseasemeth/) the UALCAN (http://ualcan.path.uab.edu/index.html), and the Wanderer (http://maplab.imppc.org/) database were used to screen for potential methylation sites in the whole sequence of GINS4 DNA and to investigate the correlations between clinicopathologic parameters of HCC patients, GINS4 expression and its methylation values.

### Screening of Genes Co-expressed With GINS4

A cluster of lncRNAs, miRNAs, mRNA, and RNA-binding protein (RBP) as well as transcription factors (TF) which interact with GINS4 were identified *via* the RAID database (RAID v2.0, www.rna-society.org/raid/) (42). The online cBioPortal database (http://www.cbioportal.org/) and GEPIA database (http://gepia.cancer-pku.cn/index.html) were queried to acquire co-expressed genes of GINS4 in HCC. Spearman correlation coefficient and Pearson correlation coefficient (PCC) were used to determine the degree of correlation between GINS4 and its co-expressed genes. R software (version 3.6.3) was used to analyze the HCC transcriptome expression matrix screened from the TCGA database to identify genes co-expressed with GINS4.

### Functional Enrichment Analysis

We performed GO and KEGG of genes co-expressed with GINS4 using “clusterProfiler” R package to predict the biological process of GINS4 in HCC (43, 44). HCC with a functional gene set were further determined *via* GSEA software downloaded from https://www.broadinstitute.org/gsea/, thus acquiring significant biological processes enriched by GINS4 (45). The pathway with a nominal P < 0.05 and FDR < 0.05 was significant (42).

### Statistical Analysis

Statistical analysis and graphic production were conducted through R language software (R 3.6.3 version). Chi-square test and Fishers exact test were applied for analyzing the correlation between GINS4 expression and clinicopathological parameters of HCC patients. Wilcoxon rank sum test were applied for comparing the GINS4 expression level in different groups. The ROC curves and corresponding AUC values were applied for determining the diagnostic significance of GINS4 and AFP levels in HCC samples in contrast to the control tissues. It was significant when P value was below 0.05.

## Results

### GINS4 Is Overexpressed in HCC and Significantly Associated With Clinicopathological Characteristics of HCC Patients

**Figure 2:**
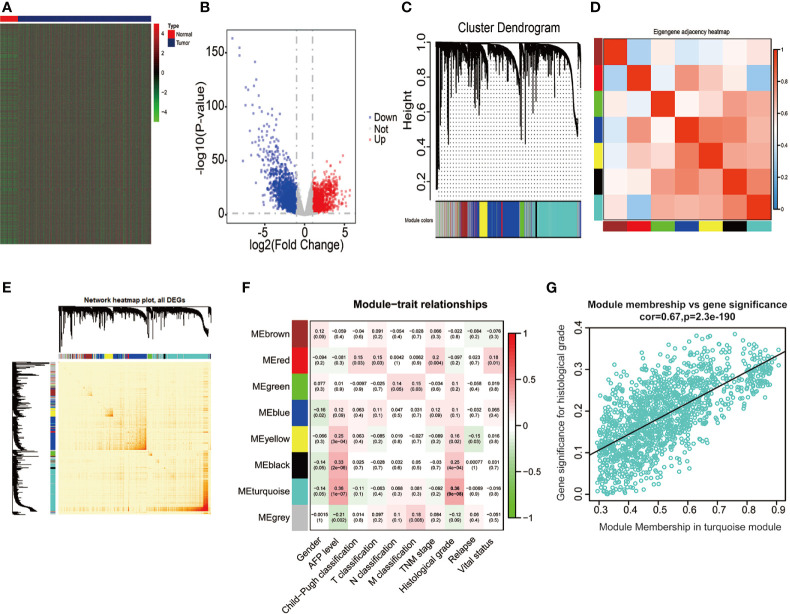
Bioinformatic analysis of HCC samples and matched normal adjacent tissues extracted from the TCGA dataset. **(A, B)** The heatmap and volcano plot of 4,344 DEGs between 374 HCC and 50 normal tissues. **(C)** Gene dendrogram of DEGs clustering and module screening according to mRNA expression profiles. **(D, E)** Heatmap of the correlation coefficient in the modules. **(F)** Correlations of module eigengenes and a series of clinical traits. **(G)** Analogous scatter plots for the turquoise module. DEGs, differently expressed genes.

We extracted the mRNA expression profiles from 50 adjacent normal liver samples and 374 HCC tissues in the TCGA database, thus unraveling 4344 DEGs (│log_2_FC│ > 1, P < 0.05, FDR < 0.05), including 2,019 upregulated DEGs (log_2_FC > 1, P < 0.05) and 2,325 downregulated DEGs (log_2_FC < −1, P < 0.05) (**Figures 2A, B**). Above 4,344 DEGs in the TCGA database were applied for conducting gene co-expression network through WGCNA approach, thus estimating pivotal and candidate mRNAs that modulated histopathological grade in the progression of HCC (46). Based on the standard scale‐free network distribution, the soft threshold power value was set as 5 (**Supplementary Figures 1A, B**). On the basis of the criterion of dynamic cut tree, the least gene number of every network and the cut-height for the integration of modules was 30 and 0.25, respectively. The correlation of characteristic genes in integrated modules was above 0.95. As revealed in **Figure 2C**, eight co-expression modules were identified among all genes *via* the Topological Overlap Matrix (TOM). The gray module indicated a gene set without significant association with any clinical characteristics. The heatmap was applied for estimating a cluster of correlated eigengenes (**Figures 2D, E**). We further evaluated the associations between MEs and clinical parameters, including gender, AFP level, Child-Pugh classification, T classification, N classification, M classification, TNM stage, histological grade, relapse, and survival status. There was the greatest correlation between the turquoise module and histological grade (r = 0.36, P < 0.0001) (**Figure 2F**), which was chosen as a module of interest to be further analyzed. GINS4 (GS = 0.3262, MM = 0.8681, P < 0.0001) was one of hub genes significantly associated with histological grade in the turquoise module (**Figure 2G**), indicating that GINS4 potentially predicts the prognosis of HCC base on histological grade.

**Figure 3:**
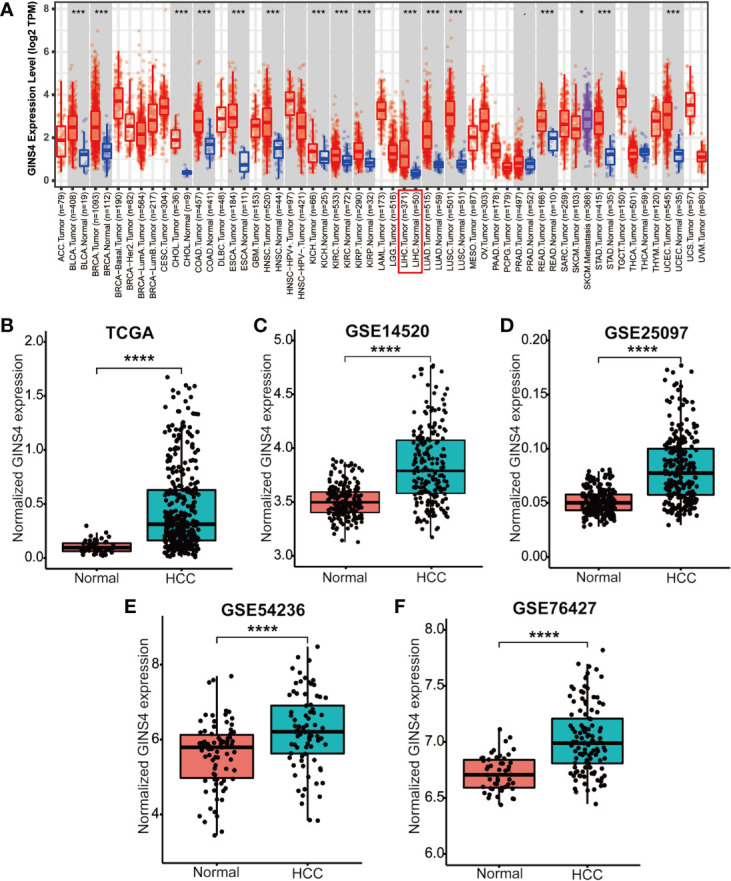
GINS4 mRNA levels are significantly upregulated in HCC. GINS4 mRNA expression in HCC and adjacent normal samples extracted from **(A)** the TIMER, **(B)** the TCGA, **(C)** GSE14520, **(D)** GSE25097, **(E)** GSE54236, and **(F)** GSE76427 database. (*P < 0.05, ***P < 0.001, and ****P < 0.0001).

**Figure 4:**
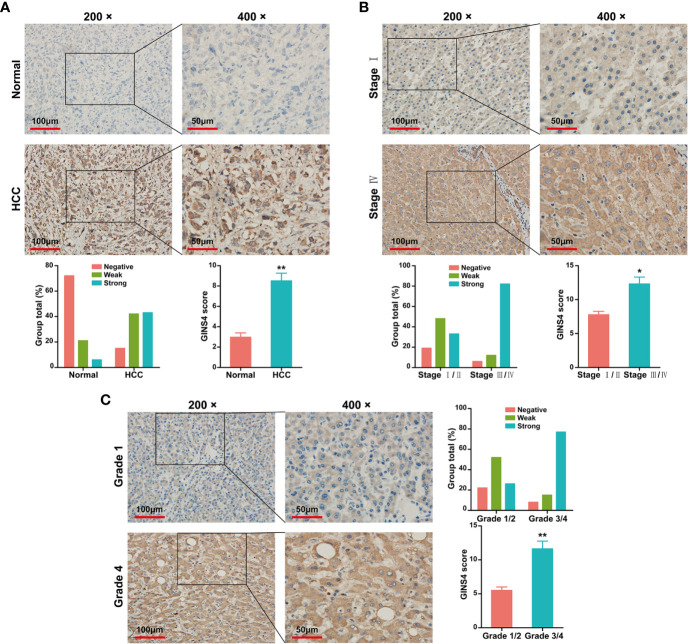
GINS4 protein is greatly expressed in HCC samples and is positively related to disease stage. IHC analysis revealed GINS4 protein level **(A)** between HCC tissues and adjacent normal liver tissues, **(B)** between TNM I/II stage and TNM III/IV stage HCC, and **(C)** between histological grade 1/2 and histological grade 3/4 HCC. (magnification, 200× and 400×; *P < 0.05 and **P < 0.01). IHC, immunohistochemistry.

Data from the Tumor Immune Estimation Resource (TIMER) database demonstrated that GINS4 mRNA expression was significantly elevated in multiple solid tumors, such as HCC, lung squamous cell carcinoma, gastric cancer, cholangiocarcinoma, and esophageal cancer (**Figure 3A**). To further identify the GINS4 level in HCC, we extracted the mRNA expression profiles from TCGA and GEO databases. The results revealed that compared with the normal liver tissue samples, GINS4 was significantly overexpressed in the HCC samples from the TCGA database (**Figure 3B**) and four GEO datasets (including GSE14520, GSE25097, GSE54236, and GSE76427) (**Figures 3C–F**). Additionally, we undertook IHC of HCC samples and matched liver tissues from 35 cases with primary HCC to further investigate GINS4 protein expression level in HCC. GINS4 protein expression was significantly increased in HCC tissues compared with adjacent normal liver samples (P < 0.01) (**Figure 4A**). Furthermore, greater GINS4 level was positively related to advanced TNM stage (TNM stage I and II *versus* TNM stage III and IV, P < 0.05) (**Figure 4B**) and worse pathological differentiation (Grade 1 and 2 *versus* Grade 3 and 4, P < 0.01) (**Figure 4C**). These results highlight that GINS4 is prominently overexpressed at both mRNA and protein levels in HCC and increased GINS4 expression potentially indicates the progression of HCC.

**Figure 5:**
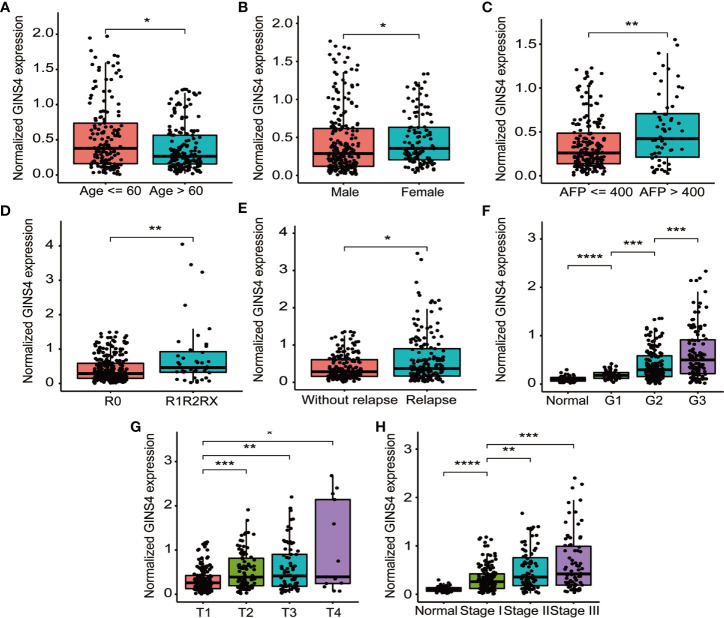
GINS4 mRNA level is significantly correlated with multiple clinicopathologic parameters in 371 HCC cases from the TCGA database. GINS4 mRNA level in HCC patients stratified by **(A)** age, **(B)** gender, **(C)** AFP level, **(D)** residual tumor, **(E)** relapse, **(F)** histologic grade, **(G)** T classification, and **(H)** TNM stage. (*P < 0.05, **P < 0.01, ***P < 0.001, and ****P < 0.0001).

**Table 1** demonstrated the correlation between GINS4 mRNA levels and clinicopathologic parameters of 371 HCC patients extracted from the TCGA. Statistical analyses suggested that GINS4 expression was significantly correlated with age (P = 0.009), gender (P = 0.001), AFP level (P = 0.049), T classification (P = 0.007), TNM stage (P = 0.011), histologic grade (P < 0.001), the status of residual tumor (P = 0.023), and relapse (P = 0.018) as well as vital status (P = 0.004). Specifically, GINS4 expression was significantly greater in HCC patients that belong to age ≤60 years old (P = 0.011), female (P = 0.010), AFP level >400 (P = 0.007), with residual tumor (P = 0.009), relapse (P = 0.017) (**Figures 5A–E**). Similarly, GINS4 mRNA level was significantly greater in histologic grade 3 HCC than histologic grade 1 HCC (P < 0.001) (**Figure 5F**). We also found progressive increase in the GINS4 expression with advanced tumor T classification and TNM stage (P < 0.0001) (**Figures 5G, H**), highlighting that GINS4 expression was positively related to the progression of HCC. In contrast, there were no correlations between GINS4 mRNA level and other clinicopathological characteristics, including the status of liver fibrosis (P = 0.5), HBV infection (P = 0.73), HCV infection (P = 0.63), vascular invasion (P = 0.26), and tumor status (P = 0.18) (**Supplementary Figures 2A–E**).

**Table 1:** Correlation between GINS4 expression and clinicopathological variables in 371 HCC patients extracted from TCGA database.

Variable	Groups	N	Low	High	χ^2^	P
Age (years)	≤60	177	76	101	6.75	**0.009**
	>60	193	110	83		
	NA	1				
Gender	Male	250	141	109	10.3	**0.001**
	Female	121	46	75		
	NA					
Race	White	186	99	87	2.38	0.304
	Asian	158	71	87		
	Black	17	8	9		
	NA	10				
Family history	No	208	97	111	0.84	0.361
	Yes	112	59	53		
	NA	51				
BMI	≤26	203	100	103	0.32	0.574
	>26	132	70	62		
	NA	36				
AFP level	≤400	213	117	96	3.87	**0.049**
	>400	65	26	39		
	NA	93				
Fibrosis score	0	74	48	26	2.96	0.398
	1-2	31	17	14		
	3-4	28	14	14		
	5-6	79	42	37		
	NA	159				
Hepatic inflammation	No	117	63	54	1.24	0.537
	Mild	99	52	47		
	Severe	18	12	6		
	NA	137				
Viral infection	HBV	97	43	54	1.88	0.17
	HCV	49	26	23		
	Both	7	3	4		
	No	199	106	93		
	NA	19				
Vascular invasion	None	206	109	97	0.68	0.713
	Micro	93	51	42		
	Macro	16	7	9		
	NA	56				
Child-Pugh classification	A	217	121	96	/	0.255**^†^**
	B	21	9	12		
	C	1	0	1		
	NA	132				
T classification	T1	181	106	75	12.27	**0.007**
	T2	94	40	54		
	T3	85	33	52		
	T4	13	5	8		
	TX	1	1	0		
	NA	2				
N classification	N0	252	124	128	/	0.623**^†^**
	N1	4	1	3		
	NX	114	62	52		
	NA	1				
M classification	M0	266	132	134	/	0.622**^†^**
	M1	4	3	1		
	MX	101	52	49		
	NA					
TNM stage	I	171	99	72	/	**0.011^†^**
	II	86	40	46		
	III	85	33	52		
	IV	5	4	1		
	NA	24				
Histologic grade	G1	55	42	13	25.47	**<0.0001**
	G2	177	93	84		
	G3	122	45	77		
	G4	12	4	8		
	NA	5				
Tumor status	With tumor	110	51	59	1.22	0.269
	Tumor free	234	125	109		
	NA	27				
Residual tumor	R0	324	172	152	/	**0.023^†^**
	R1	17	4	13		
	R2	1	1	0		
	RX	22	7	15		
	NA	7				
Relapse	No	191	103	88	5.6	**0.018**
	Yes	180	74	106		
	NA					
Vital status	Dead	130	48	82	8.46	**0.004**
	Alive	240	128	112		
	NA	1				

^†^means the P value of Fisher’s exact test conducted on the condition of small sample.

NA, not available. The bold values mean the clinical variable with statistically significance..

### GINS4 Can Effectively Distinguish HCC Patients From Nontumor Individuals

To identify the diagnostic significance of GINS4 in HCC, ROC curve was applied for analyzing the AUC of GINS4 expression stratified by clinical variables of HCC patients in the TCGA database. As revealed in **Supplementary Figure 3A**, GINS4 could effectively distinguish normal liver samples from HCC samples with an AUC of 0.865 (95% CI = 0.828–0.903). Additionally, the AUC value for the capacity of GINS4 expression level to differentiate HCC samples at TNM I, II, and III stage from adjacent tumor tissues was 0.835 (95% CI = 0.796–0.896), 0.878 (95% CI = 0.822–0.937), and 0.906 (95% CI = 0.840–0.946), respectively (**Supplementary Figures 3B–D**). Thus, our findings revealed that GINS4 displays a favorable ability to distinguish HCC patients and healthy individuals, even for early-stages HCC.

**Figure 6:**
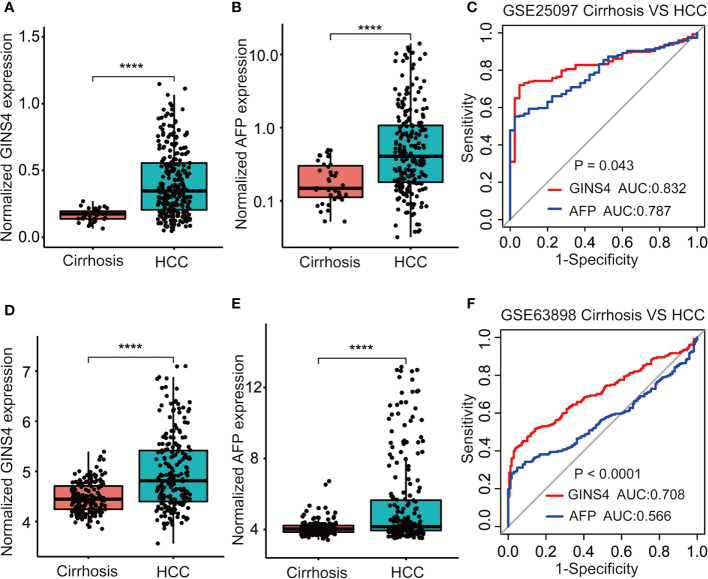
GINS4 harbors a great performance to differentiate HCC from liver cirrhosis. GINS4 mRNA level between liver cirrhosis and HCC samples from **(A)** GSE25097 and **(D)** GSE63898. AFP mRNA level between liver cirrhosis and HCC samples from **(B)** GSE25097 and **(E)** GSE63898. ROC curve demonstrates the discriminative efficiency of GINS4 and AFP between liver cirrhosis and HCC samples from **(C)** GSE25097 and **(F)** GSE63898. (****P < 0.0001). ROC, the receiver operating characteristic.

Furthermore, we formulated ROC curves to differentiate HCC patients from liver cirrhosis cases extracted from the GSE25097 and GSE63898 databases. In the GSE25097 dataset, GINS4 and alpha-fetoprotein (AFP) mRNA expression were both prominently greater in HCC than liver cirrhosis tissues (**Figures 6A, B**). The AUC of GINS4 (0.832, 95% CI = 0.781–0.882) was higher than that of AFP (0.787, 95% CI = 0.729–0.845) (P = 0.043) (**Figure 6C**). As for data from the GSE63898 dataset, GINS4 and AFP expression in HCC were remarkably elevated compared with liver cirrhosis (**Figures 6D, E**). The AUC of 0.708 (95% CI = 0.658–0.758) for GINS4 was significantly higher than that of 0.566 (95% CI = 0.510–0.622) for AFP (P < 0.0001) (**Figure 6F**). Our findings indicate that GINS4 is endowed with a relatively accurate performance to differentiate HCC from liver cirrhosis.

**Figure 7:**
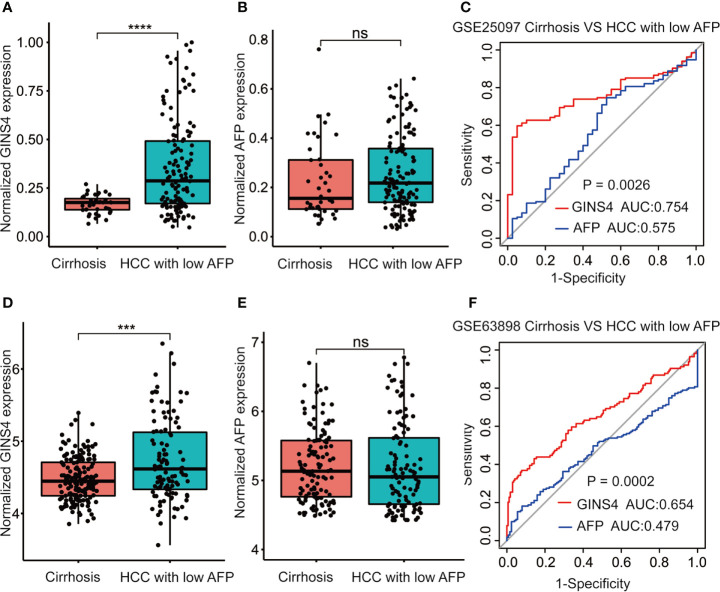
GINS4 harbors a satisfactory performance to distinguish low AFP-expressing HCC from liver cirrhosis. GINS4 expression level between liver cirrhosis and low AFP-expressing HCC from **(A)** GSE25097 and **(D)** GSE63898. AFP expression level between liver cirrhosis and low AFP-expressing HCC from **(B)** GSE25097 and **(E)** GSE63898. ROC curve reveals the capability of GINS4 and AFP in differentiating liver cirrhosis and low AFP-expressing HCC from **(C)** GSE25097 and **(F)** GSE63898. (***P < 0.001, ****P < 0.0001). ns, no significance; ROC, the receiver operating characteristic.

We further assessed the discriminative performance of GINS4 between low AFP-expressing HCC individuals and liver cirrhosis patients from the GSE25097 and GSE63898 datasets. GINS4 expression was significantly greater in HCC cases with low AFP expression than liver cirrhosis patients from above two datasets (**Figures 7A, D** **)**. Conversely, there were no statistically significant difference in AFP level between liver cirrhosis and such HCC in two datasets (**Figures 7B, E**). ROC curve demonstrated that the AUC value for GINS4 was significantly higher compared with that for AFP in both datasets (0.754, 95% CI = 0.683–0.826 *versus* 0.575, 95% CI = 0.472–0.678, P = 0.0026 in GSE25097, **Figure 7C**; 0.654, 95% CI = 0.586–0.723 *versus* 0.479, 95% CI = 0.413–0.544, P = 0.0002 in GSE63898, **Figure 7F**). Above results highlight that GINS4 potentially serves as an instrument to screen low AFP-expressing HCC individuals.

### Increased GINS4 Expression Predicts Unfavorable Prognosis in HCC Patients

**Figure 8:**
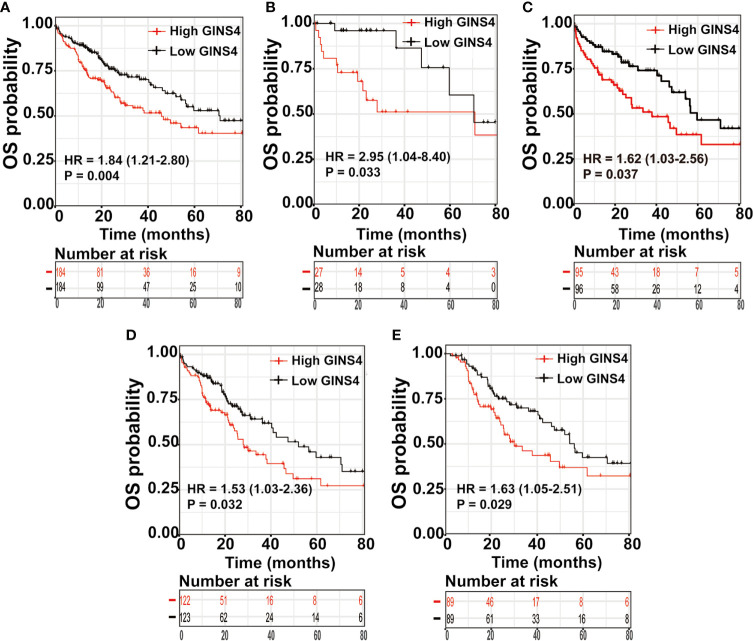
High GINS4 expression is a significantly adverse prognostic factor in HCC patients. Survival analysis shows that elevated GINS4 expressions predicts poor prognosis in **(A)** all HCC patients from the TCGA database, **(B)** HCC patients at histological grade 1, **(C)** HCC patients with age >60 years old, **(D)** HBV infection-negative HCC patients, and **(E)** HCC subgroup with relapse. OS, overall survival.

We further estimated the prognostic significance of GINS4 in HCC through the Kaplan‐Meier curve. As revealed in **Figure 8A**, among all HCC patients, high CINS4- expressing HCC patients exhibited more unfavorable clinical outcome than those with low CINS4 expression (HR = 1.84, 95% CI = 1.21–2.8, P = 0.0038). We further investigated the correlation between GINS4 mRNA level and OS of HCC patients stratified by a variety of clinicopathologic features. Specifically, for HCC patients at histological grade 1, high GINS4-expressing patients were characteristic with worse OS compared with those with low GINS4 level (HR = 2.95, 95% CI = 1.04–8.40, P = 0.033) (**Figure 8B**). Additionally, high GINS4-expressing HCC patients belonging to age >60 years old (HR = 1.62, 95% CI = 1.03–2.56, P = 0.037), HBV infection-negative (HR = 1.53, 95% CI = 1.03–2.36, P = 0.032), and occurring relapse (HR = 1.63, 95% CI = 1.05–2.51, P = 0.029) subgroups were with significantly diminished OS than low GINS4-expressing HCC (**Figures 8C–E**).

Furthermore, we formulated univariate and multivariate Cox analyses to estimate the prognostic significance of GINS4 expression in HCC. In the univariate analysis, viral hepatitis infection, vascular invasion, T classification, M classification, TNM stage, tumor status, residual tumor, and GINS4 expression were significantly associated with the prognosis of HCC (P < 0.05) (**Table 2**). Above parameters were all incorporated into the multivariate Cox analyses. As shown in **Table 2**, multivariate Cox proportional hazards model revealed that high GINS4 expression (HR = 1.46, 95% CI = 1.01–2.1, P = 0.043) and advanced TNM stage (HR = 1.27, 95% CI = 1.01–1.62 for TNM stage II, P = 0.045; HR = 2.56, 95% CI = 1.66–3.96 for TNM stage III, P < 0.001) were independent unfavorable prognostic factors for the OS of HCC.

**Table 2:** Univariate and multivariate analysis for OS of 371 HCC patients from the TCGA database.

c			Univariate analysis		Multivariate analysis	
Variables	Mean survival months	95% CI	HR (95% CI)	P	HR (95% CI)	P
Total	26.7	24.2–29.2				
**Age (years)**						
≤60	26.5	22.9–30	Ref			
>60	26.9	23.4–30.4	1.25 (0.88–1.77)	0.211		
**Gender**						
Female	28.1	23.4–32.8	Ref			
Male	26	23.1–28.9	0.81 (0.57 –1.16)	0.257		
**Race**						
White	28.2	24.5–31.9	Ref			
Asian	25.9	22.3–29.6	0.77 (0.53–1.13)	0.179		
Black	20.7	10.7–30.7	1.19 (0.52–2.74)	0.681		
**Family history**						
No	26.2	22.9–29.6	Ref			
Yes	29.3	24.9–33.7	1.18 (0.82–1.70)	0.379		
**BMI**						
≤26	26.3	23–29.7	Ref			
>26	29.2	24.9–33.5	0.95 (0.65–1.39)	0.786		
**AFP level**						
≤400	28.8	25.6–32	Ref			
>400	31.4	24.3–38.5	1.06 (0.65–1.73)	0.827		
**Fibrosis score**						
0	36.4	29.1–43.6	Ref			
1–4	28.6	23.2–33.9	0.80 (0.42–1.51)	0.496		
5–6	29.2	23.8–34.5	0.76 (0.42–1.36)	0.352		
**Hepatic inflammation**						
None	35.9	30.6–41.2	Ref			
Mild	26.8	22.5–31.1	1.25 (0.75–2.08)	0.399		
Severe	31.2	16.6–45.8	1.12 (0.44–2.87)	0.811		
**Viral infection**						
Yes	25.2	21.7–28.6	Ref			
No	28.7	25–32.4	0.5 (0.34–0.73)	< 0.001	0.62 (0.21–1.84)	0.39
**Vascular invasion**						
None	29.1	25.6–32.5	Ref			
Micro	25.2	20.3–30.2	1.21 (1.01–1.54)	0.042	0.89 (0.51–1.56)	0.687
Macro	21.6	13.7–29.5	2.24 (1.07–4.72)	0.033	1.47 (0.64–3.35)	0.363
**Child-Pugh classification**						
A	30.3	26.9–33.6	Ref			
B	25.2	14.2–36.3	1.57 (0.75–3.31)	0.235		
C	54.1	\	2.09 (0.29–15.19)	0.467		
**T classification**						
T1	29.5	25.9–33.2	Ref			
T2	25.9	20.9–30.8	1.44 (1.05–1.98)	0.024	0.74 (0.17–3.19)	0.687
T3	23.3	17.8–28.8	2.62 (1.72, 3.97)	<0.001	2.7 (0.68–10.72)	0.158
T4	15.1	9.4–20.9	5.29 (2.64, 10.6)	<0.001	3.79 (0.94–15.19)	0.06
**N classification**						
N0	28.8	25.5–32	Ref			
N1	19.4	0–46.1	2.11 (0.52–8.62)	0.298		
NX	22.2	18.6–25.8	1.3 (0.67–2.50)	0.433		
**M classification**						
M0	28.1	25–31.2	Ref			
M1	14.3	0–35.8	4.28 (1.34–13.6)	0.014	4.03(0.97–15.67)	0.996
MX	23.5	19.8–27.2	1.61 (1.11–2.33)	0.013	1.36 (0.76–2.44)	0.296
**TNM stage**						
I	29.8	26–33.6	Ref			
II	25.8	20.4–31.1	1.52 (1.02–2.32)	0.048	1.27 (1.01–1.62)	**0.045**
III	23	17.9–28.2	2.68 (1.75–4.08)	<0.001	2.56 (1.66–3.96)	**<0.001**
IV	11.5	0–28	5.5 (1.70–17.8)	0.005	0	0.997
**Histologic grade**						
G1	30.1	22.8–37.3	Ref			
G2	26.2	22.6–29.9	1.18 (0.70–2.00)	0.541		
G3	26.2	22–30.5	1.23 (0.71–2.14)	0.457		
G4	20.8	13.1–28.5	1.69 (0.62–4.58)	0.301		
**Tumor status**						
Tumor free	24.1	21.1–27	Ref			
With tumor	34.3	29.5–39	1.59 (1.11–2.28)	0.012	1.28 (0.87–1.88)	0.203
**Residual tumor**						
R0	28.3	25.6–31	Ref			
R1	27.7	15.2–40.1	1.42 (1.01–2.02)	0.044	0.7 (0.31–1.58)	0.394
R2	7.4	\	10.51(1.43–77.0)	0.021	5.57(0.34–91.07)	0.229
RX	8.7	4.8–12.5	3.07 (1.48–6.39)	0.003	3.39 (0.95–12.1)	0.06
**Relapse**						
No	22.2	18.8–25.5	Ref			
Yes	31.5	27.9–35.1	1.34 (0.93–1.91)	0.115		
**GINS4 expression**						
low	29.7	26–33.4	Ref			
high	23.7	20.4–27	1.55 (1.1–2.2)	0.013	1.46 (1.01–2.1)	**0.043**
						

HR, hazard ratio; 95% C,: 95% confidence interval; OS, overall survival; Ref, reference. The bold values mean the clinical variable with statistically significance.

### Development and Validation of Nomogram Model

**Figure 9:**
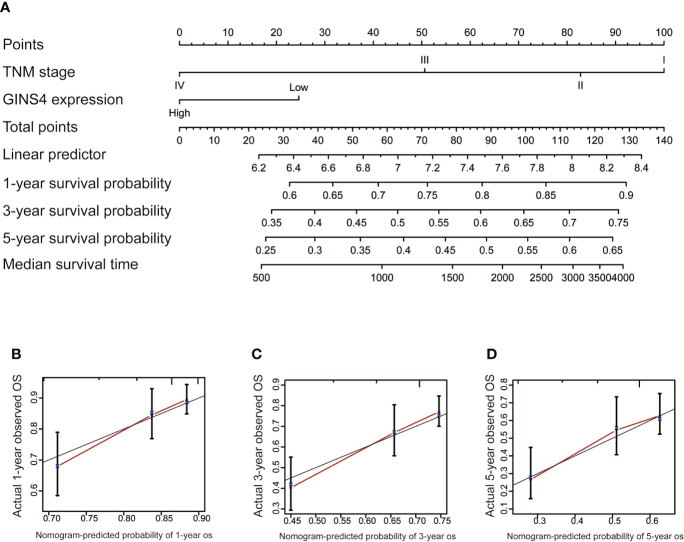
Nomogram to predict the long-term OS of 371 HCC patients from the TCGA database. **(A)** Nomogram model including TNM stage and GINS4 expression level to predict the 1-, 3-, and 5-year OS of HCC patients. The calibration plots of **(B)** 1-year, **(C)** 3-year, and **(D)** 5 year-OS of HCC patients. OS, overall survival.

To estimate the long-term survival of HCC individuals, we included all significant independent prognostic factors identified by the multivariate analyses, thus formulating a user-friendly nomogram with a C-index of 0.724. As revealed in **Figure 9A**, TNM stage and GINS4 mRNA level made great contributions to clinical outcome of HCC. The calibration plots for the OS probability of 1-year, 3-year, or 5-year in HCC patients showed an optimal consistency between nomogram prediction and practical observation (**Figures 9B–D**).

**Figure 10:**
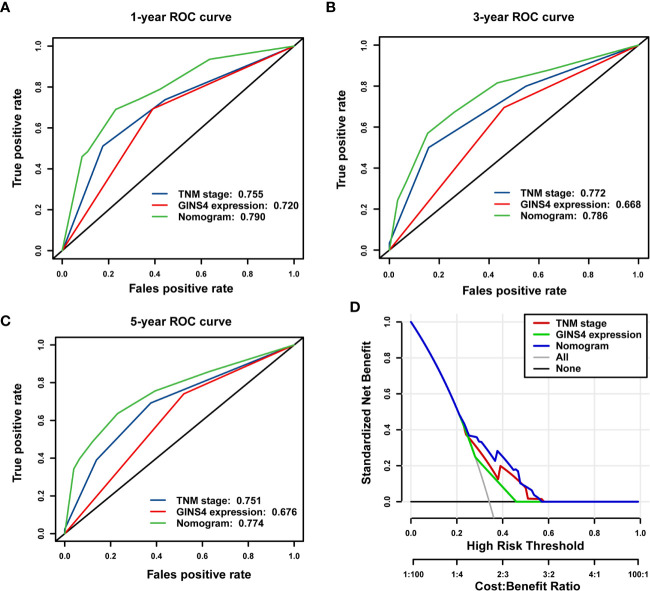
The nomogram model shows a relatively desirable predictive accuracy and clinical utility to predict OS of 371 HCC patients from the TCGA database. The time- dependent ROC demonstrated the AUC values of the nomogram, TNM stage, and GINS4 expression level at **(A)** 1-year, **(B)** 3-year, and **(C)** 5-year OS of HCC patients. **(D)** DCA for evaluating the clinical utility of nomogram model, TNM stage, and GINS4 expression level in predicting the OS of HCC cases. The x-axis revealed the range of threshold probabilities and the y-axis showed the NB. The gray line farthest left indicated all strategies. The horizontal black line represented none strategy. OS, overall survival; ROC, the receiver operating characteristic; DCA, Decision curve analysis; AUC, the area under the curve; NB, net benefit.

The time-ROC curves were further employed for nomogram validation. The 1-year OS AUC of the nomogram model, TNM stage, and GINS4 expression level was 0.790 (95% CI = 0.710–0.855), 0.755 (95% CI = 0.673–0.826), and 0.720 (95% CI = 0.657–0.783), respectively (**Figure 10A**). Similarly, the nomogram model showed the highest 3-year OS AUC of 0.786 (95% CI 0.713–0.856), followed by TNM stage (AUC = 0.772, 95% CI = 0.715–0.842) and GINS4 expression level (AUC = 0.668, 95% CI = 0.601–0.719) (**Figure 10B**). The AUC at 5 years of the nomogram was 0.774 (95% CI = 0.661–0.877), significantly more discriminative than that of TNM stage being 0.751 (95% CI = 0.661–0.820) and that of GINS4 expression level being 0.676 (95% CI = 0.578–0.728) (**Figure 10C**). Thus, our nomogram comprising TNM stage and GINS4 expression level displayed a relatively satisfactory predictive accuracy for the long-term prognosis of HCC.

Furthermore, DCA was used to compare the clinical usefulness of nomogram with that of TNM stage and GINS4 expression level based on the threshold probability. **Figure 10D** revealed that the nomogram model exhibited a greater NB across a wider range of threshold probabilities for predicting long-term OS of HCC patients in the TCGA cohort, followed by TNM stage and GINS4 expression level. Specifically, the patients with OS probability between 0.23 and 0.58 would reap the highest NB if they selected the nomogram model. It also showed that TNM stage indicator would be applicable if OS probability of a patient was within the range of 0.25 to 0.59. Similarly, when OS probability of HCC patients was less than 0.28 or more than 0.45, decisions based on the GINS4 expression level would be meaningless. Therefore, above findings highlight that the nomogram is an excellent predicted evaluation model and it was superior to TNM stage or GINS4 expression level alone.

### GINS4 Methylation Level Is Significantly Decreased in HCC Patients

**Figure 11:**
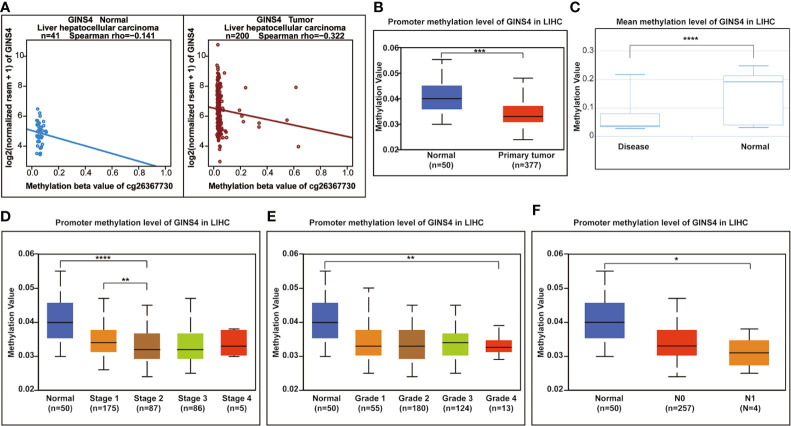
The correlation between GINS4 methylation and clinical characteristics of HCC patients. **(A)** The association between GINS4 expression and its methylation level of cg26367730 methylation site in HCC samples and matched normal liver tissues from the Wanderer. Total GISN4 methylation in HCC samples was significantly lower compared with the normal liver samples evaluated by **(B)** UALCAN and **(C)** DiseaseMeth databases. The association between GINS4 methylation levels and **(D)** TNM stage, **(E)** histological grade, and **(F)** lymph node status of HCC patients downloaded from UALCAN database.

Hypermethylation of CpG sites in promoters frequently results in transcriptional silencing. Conversely, hypomethylation of CpG sites in a gene body generally triggers an enhancive gene expression. A range of tumors are associated with promoter-specific hypomethylation and accompanied gene overexpression (47, 48). We selected the methylation site cg26367730 from the Wanderer database as the most statistically significant candidate site (**Supplementary Table 1**). As revealed in **Figure 11A**, GINS4 expression gradually decreased with incremental DNA methylation level in both adjacent normal liver samples and HCC tissues from the Wanderer database (P < 0.05), indicating that there is a potential negative association between the transcript expression of GINS4 and a number of CpG sites. Additionally, data from the UALCAN databases and the DiseaseMeth databases demonstrated that the total methylation value of GINS4 in the HCC samples was significantly decreased than normal liver samples (P < 0.001) (**Figures 11B, C**). Subsequently, we explored the correlation between GINS4 methylation level and clinicopathologic parameters of HCC patients from the UALCAN database. The lower methylation values of GINS4 in HCC patients were significantly associated with advanced TNM stage (P < 0.01), poorer pathological differentiation (P < 0.01), and lymph node metastasis (P < 0.05) (**Figures 11D–F**). Thus, above results highlighted that DNA hypomethylation, a primary epigenetic modification, potentially triggers GINS4 overexpression at the transcriptional level, thus exerts crucial effects on carcinogenesis and progression of HCC.

### High GINS4 Expression Positively Modulates Cell Cycle and PI3K/AKT/mTOR Signaling Pathway in HCC

**Figure 12:**
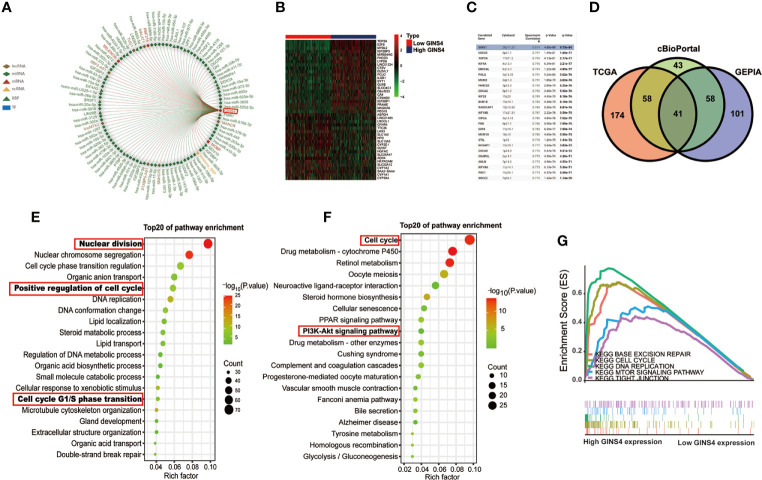
High GINS4 expression positively modulates the cell cycle pathway in HCC development. **(A)** RAID database revealed certain miRNAs, lncRNAs, RBPs, TFs interacting with GINS4. **(B)** Heatmap of the DEGs co-expressed with GINS4 in HCC samples (│log_2_FC│> 2). **(C)** The top 25 genes associated with GINS4 transcript level in HCC based on the cBioPortal database. **(D)** Venn diagram illustrated the intersection of genes co-expressed with GINS4 in HCC among the TCGA, the cBioPortal, and the GEPIA database. The top 20 signaling pathways in HCC *via* **(E)** GO and **(F)** KEGG analysis. **(G)** GSEA of the whole DEGs co-expressed with GINS4 in HCC. DEGs, differently expressed genes; TF, transcription factors; NES, normalized enrichment score; RBP, RNA-binding protein.

As revealed in **Figure 12A**, GINS4 could interacted with six lncRNAs (TUG1, MALAT1, MANCR, SNHG16, STXBP5-AS1, lincMTX2, and SBF2-AS1) and multiple RBPs (including UPF1, YTHDF2, DGCR8, QK1, FMR1, and SRSF1). We also classified all HCC patients in the TCGA database into high and low GINS4 expression groups in accordance with the median GINS4 level. There was a total of 42 DEGs (│log_2_FC│>2) between above two groups, including 22 upregulated DEGs (log_2_FC > 2) and 20 downregulated DEGs (log_2_FC < −2), which co-expressed with GINS4 in HCC samples (**Figure 12B**). The top 200 co-expressed genes of GINS4 were obtained from the cBioPortal dataset (Spearman correlation coefficient ≥0.618, P value ≤6.26e-40) (**Figure 12C**). Meanwhile, the GEPIA database was applied to screen the top 200 genes co-expressed with GINS4 (PCC ≥ 0.62) (**Supplementary Table 2**). We cross-referenced the co-expressed genes from the above three databases to obtain a total of 41 common GINS4 co-expressed genes (**Figure 12D**).

We further conducted functional analysis of co‐expressed genes to investigate the biological classification of GINS4 in HCC. The top 20 biological process (BP) concerning the significantly enriched GO terms showed that these DEGs were primarily involved in the processes of nuclear division, positive regulation of cell cycle and DNA replication as well as cell cycle G1/S phase transition (**Figure 12E**), suggesting that GINS4 potentially facilitates HCC growth and proliferation through accelerating G1/S phase transition. Additionally, KEGG pathway analysis demonstrated that enrichment results were significantly correlated with the cell cycle and phosphoinositide 3 kinase (PI3K)- protein kinase B (AKT) signaling pathway (**Figure 12F**). The GSEA results revealed that the overexpression of GINS4 positively associated with the cell cycle [normalized enrichment score (NES) = 2.112, P < 0.0001], DNA replication (NES = 1.897, P = 0.002), base excision repair (NES = 1.953, P = 0.002), and tight junction (NES = 1.726, P = 0.048) as well as mechanistic target of rapamycin (mTOR) signaling pathway (NES = 1.679, P = 0.009) (**Figure 12G**).

Pearson correlation analysis further demonstrated that upregulation of GINS4 in HCC was significant positively correlated with the expression of phosphoinositide-3-kinase, catalytic, betapolypeptide (PIK3CB, known as the coding gene of PI3K P110 subunit, R^2^ = 0.35, P < 0.0001), AKT1 (known as the coding gene of AKT subunit, R^2^ = 0.23, P < 0.0001) and MTOR (R^2^ = 0.27, P < 0.0001) as well as CCND1 (known as the coding gene of Cyclin D1 that can promote the G1/S phase transition of mitosis, R^2^ = 0.16, P < 0.0001) (**Supplementary Figures 4A–D**). Thus, these studies indicated that GINS4 potentially participates in the regulation of PI3K/AKT/mTOR and cyclin D1 level, thus facilitating the occurrence and progression of HCC.

## Discussion

HCC is a highly malignant tumor characterized with unfavorable clinical outcome and extremely high rates of mortality. Therefore, further investigation of HCC oncogenes is conducive to disclose novel and promising prognostic biomarkers and druggable targets, thus improving the clinical outcome of HCC. GINS4, a component of GINS complex, has been demonstrated a series of crucial functions in the biological process, including positive modulating in the initiation and prolongation of DNA replication, accelerating the transition of the cell cycle G1/S phase in eukaryotic cells, conferring protection against DNA damage in in both normal cells and cancer cells (20, 49–52). Significantly increased GINS4 level has been revealed in a series of human cancers, such as CRC (19, 20), NSCLC (22), gastric cancer (23), bladder cancer (21), and pancreatic cancer (24), highlighting the pivotal role of GINS4 in tumorigenesis. Nevertheless, the effect of GINS4 on HCC is relatively indistinct. Herein, our study was designed to identify the expression and the clinical and biology significance of GINS4 in HCC.

In our report, we conducted WGCNA co-expression network and revealed that GINS4 was one of hub DEGs most relevant to histological grade of HCC. GINS4 was overexpressed in HCC samples, and the expression level of GINS4 was significantly positively correlated with TNM stage and histological grade, indicating that GINS4 is an oncogene of HCC. ROC curves also demonstrated that GINS4 expression level could effectively distinguish HCC patients from non-tumor individuals (such as healthy controls and patients with liver cirrhosis). Additionally, the upregulation of GINS4 was associated with poor prognosis of HCC, especially in age >60 years old, histological grade G1, HBV-negative infection, and with recurrence subgroups, suggesting that GINS4 was a potentially independent risk factor affecting OS in HCC patients. The diagnostic and prognostic significance of GINS4 in other human tumors has also been confirmed. For example, the IHC results on tissue microarrays of 106 CRC patients revealed that enhanced GINS4 expression was positively related to advanced T stage, advanced TNM stage, and poor pathological differentiation (20). Additionally, multivariate analysis showed that GINS4 expression level in lung cancer was independent of clinical risk factors, such as gender, smoking, tumor differentiation, and tumor size, whereas it was associated with TNM stage and lymph node metastasis. The Kaplan-Meier curve also presented that high GINS4 expression predicted undesirable prognosis of all lung cancer patients and lung adenocarcinoma cases. Notably, there was no statistically significant correlation between GINS4 level and the survival of patients with lung squamous cell carcinoma (22). Similarly, gastric cancer patients with strongly positive GINS4 staining were characterized with shorter OS and DFS, suggesting that GINS4 may be a promising molecular target in the diagnosis and therapy of gastric cancer (23).

Furthermore, we found that GINS4 potentially positively modulated the cell cycle in HCC through accelerating the transition of mitotic G1/S phase and participated in malignant progression *via* PI3K/AKT/mTOR pathway based on GO and KEGG analysis. Pearson correlation analysis also demonstrated the significantly positive correlation between GINS4 mRNA and PI3KCB, AKT1, MTOR, and CCND1 transcriptome levels. PI3K/AKT/mTOR pathway is frequently activated in various human cancers, contributing to diversiform oncogenic transformation processes (such as stimulation of proliferation, survival, metabolic reprogramming, metastasis, and inhibition of apoptosis, autophagy, and aging) (53–55). Specifically, GINS4 could directly activate PI3K/AKT and MAPK/ERK pathways, thus accelerating cell proliferation and apoptosis in gastric cancer and CRC (20, 23). AKT is upregulated in 71% of HCC samples, thus accelerating the progressive growth of HCC. The activation of mTOR signaling is also revealed in above 48% of HCC samples and is related to undesirable prognosis in HCC therapy (55). As a pivotal cell cycle regulator, CyclinD1 is essential for accelerating the G1/S phase transition. CCND1, the coding gene of CyclinD1, has also been identified as a candidate proto-oncogene. The amplification and overexpression of CCND1 can alter the progression of the cell cycle and may be involved in the occurrence of tumors (56). Notably, Krüppel-like factor 4 (KLF4) diminishes GINS4 expression through binding to the promoter of GINS4, thus suppressing the development of CRC (20). Lymphoid-specific helicase (LSH) stabilizes and enhances GINS4 expression *via* binding to 3’UTR region of GINS4, thus facilitating lung cancer development (22). IL-6-induced the upregulation of DNA-methyltransferase (DNMT) inhibits miR-370, leading to high GINS4 expression and tumor growth in bladder cancer (21). Thus, suppression of GINS4 potentially represents a novel strategy to retard tumor development.

In conclusion, GINS4 is upregulated in HCC and high expression of GINS4 is significantly related to shorter survival in HCC patients. GINS4 may positively modulate the cell cycle process of HCC and potentially trigger the tumorigenesis and progression of HCC in a PI3K/AKT/mTOR dependent manner, which needs to be further experimental verification.

## Data Availability Statement 

The original contributions presented in the study are included in the article/**Supplementary Material**. Further inquiries can be directed to the corresponding author.

## Ethics Statement

The studies involving human participants were reviewed and approved by the medical ethics committee of the Third Xiangya Hospital. The patients/participants provided their written informed consent to participate in this study.

## Author Contributions

PGC and ZZ designed/planned the study and wrote the paper. ZZ and PC performed experiment operation and computational modeling, and acquired and analyzed data. ZZ, PC, and HX performed imaging analysis. ZZ, PC, HX, and PGC participated in discussion of related data. ZZ drafted the manuscript. All authors contributed to the article and approved the submitted version.

## Funding

This work was supported by the National Natural Science Foundation of China (81872473) and the Hunan Province Science and Technology plan (2017SK2052).

## Conflict of Interest

The authors declare that the research was conducted in the absence of any commercial or financial relationships that could be construed as a potential conflict of interest.

## Supplementary Material

Supplementary Figure 1 Analysis of network topology for multiple soft-thresholding powers. **(A)** The scale-free fit index and **(B)** the mean connectivity of multiple soft-thresholding powers.

Supplementary Figure 2 GINS4 mRNA expression in HCC patients stratified by multiple clinicopathologic parameters, including **(A)** liver fibrosis, **(B)** HBV infection, **(C)** HCV infection, **(D)** vascular invasion, **(E)** tumor status.

Supplementary Figure 3 GINS4 exhibits a promising capability to discriminate HCC patients from healthy individuals from the TCGA database. ROC curve for **(A)** all HCC patients, **(B)** HCC patients at TNM I stage, **(C)** HCC patients at TNM II stage, and **(D)** HCC patients at TNM III stage. ROC, the receiver operating characteristic.

Supplementary Figure 4 GINS4 transcriptome level is positively correlated with genes associated with PI3K/AKT/mTOR pathway in 371 HCC patients from the TCGA database. Pearson correlation analysis revealed the correlation between GINS4 mRNA level and **(A)** PIK3CB, **(B)** AKT1, **(C)** MTOR, and **(D)** CCND1.
