# Supplementary figures and images for: Overexpression of GINS4 Is Associated With Tumor Progression and Poor Survival in Hepatocellular Carcinoma
Source: Front Oncol. 2021 Mar 25;11:654185. doi: 10.3389/fonc.2021.654185 (PMC8027117; doi:10.3389/fonc.2021.654185)

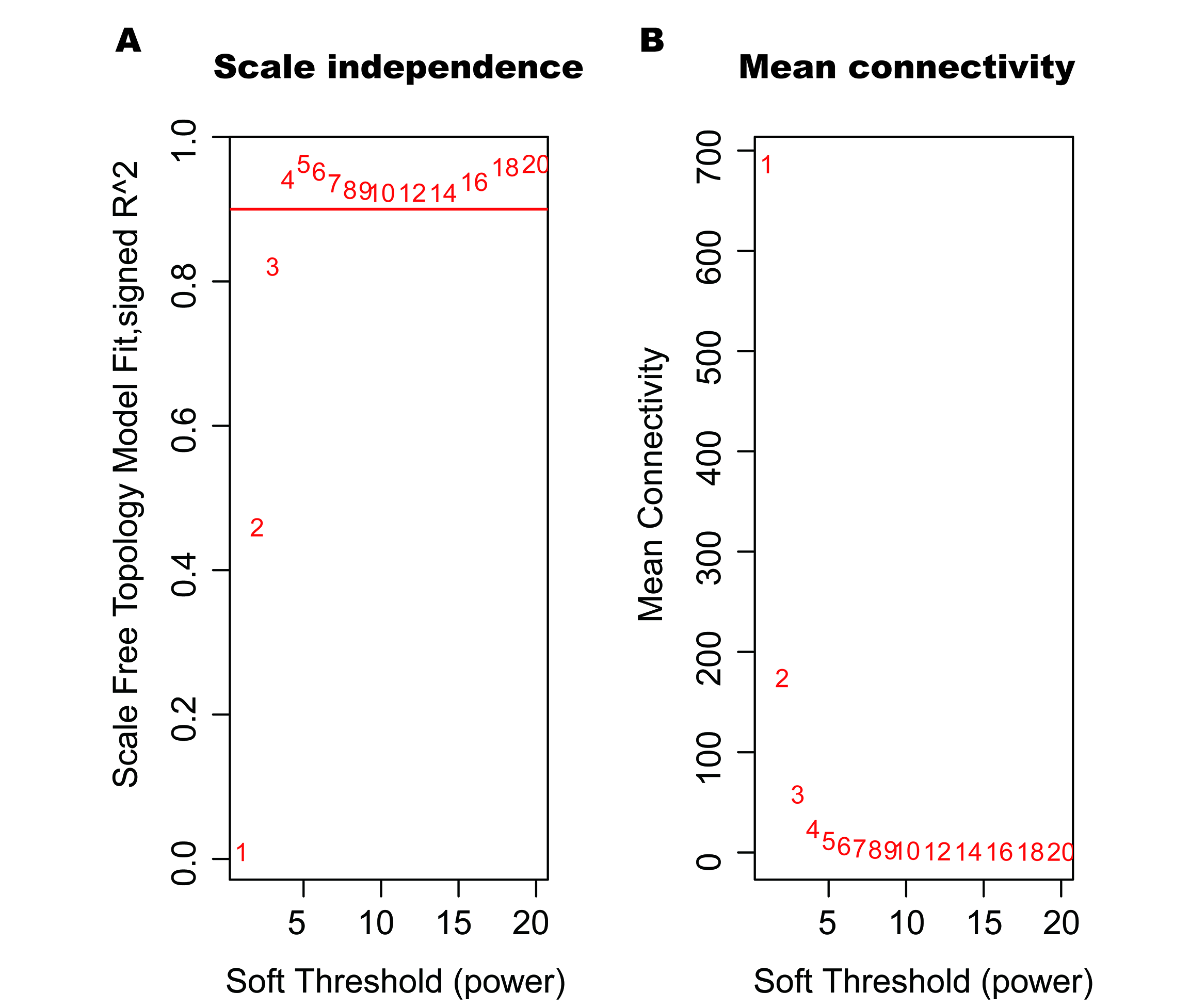

Supplement: Supplementary Figure 1 — Analysis of network topology for multiple soft-thresholding powers. (A) The scale-free fit index and (B) the mean connectivity of multiple soft-thresholding powers. [file Image_1.tif]

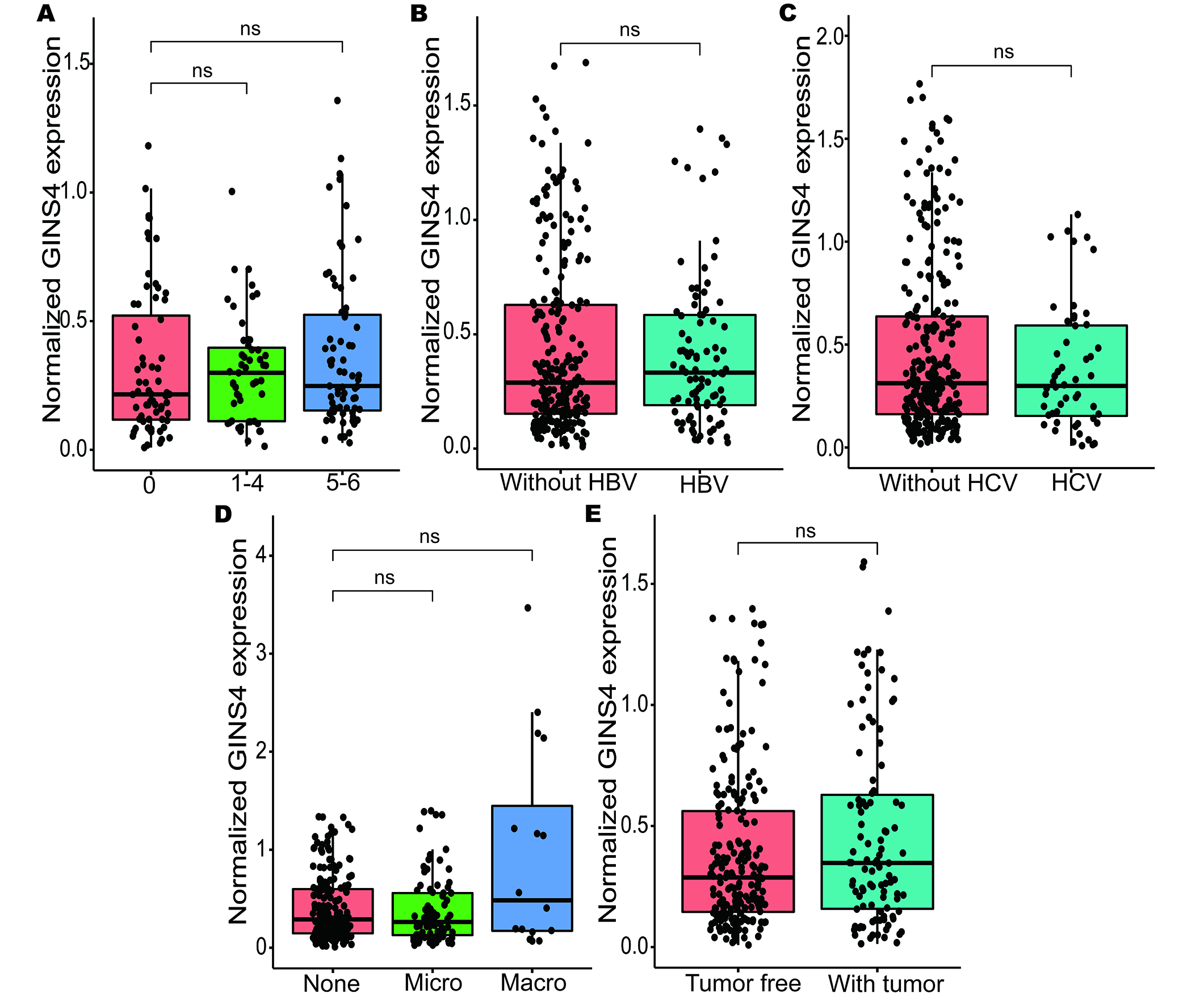

Supplement: Supplementary Figure 2 — GINS4 mRNA expression in HCC patients stratified by multiple clinicopathologic parameters, including (A) liver fibrosis, (B) HBV infection, (C) HCV infection, (D) vascular invasion, (E) tumor status. [file Image_2.tif]

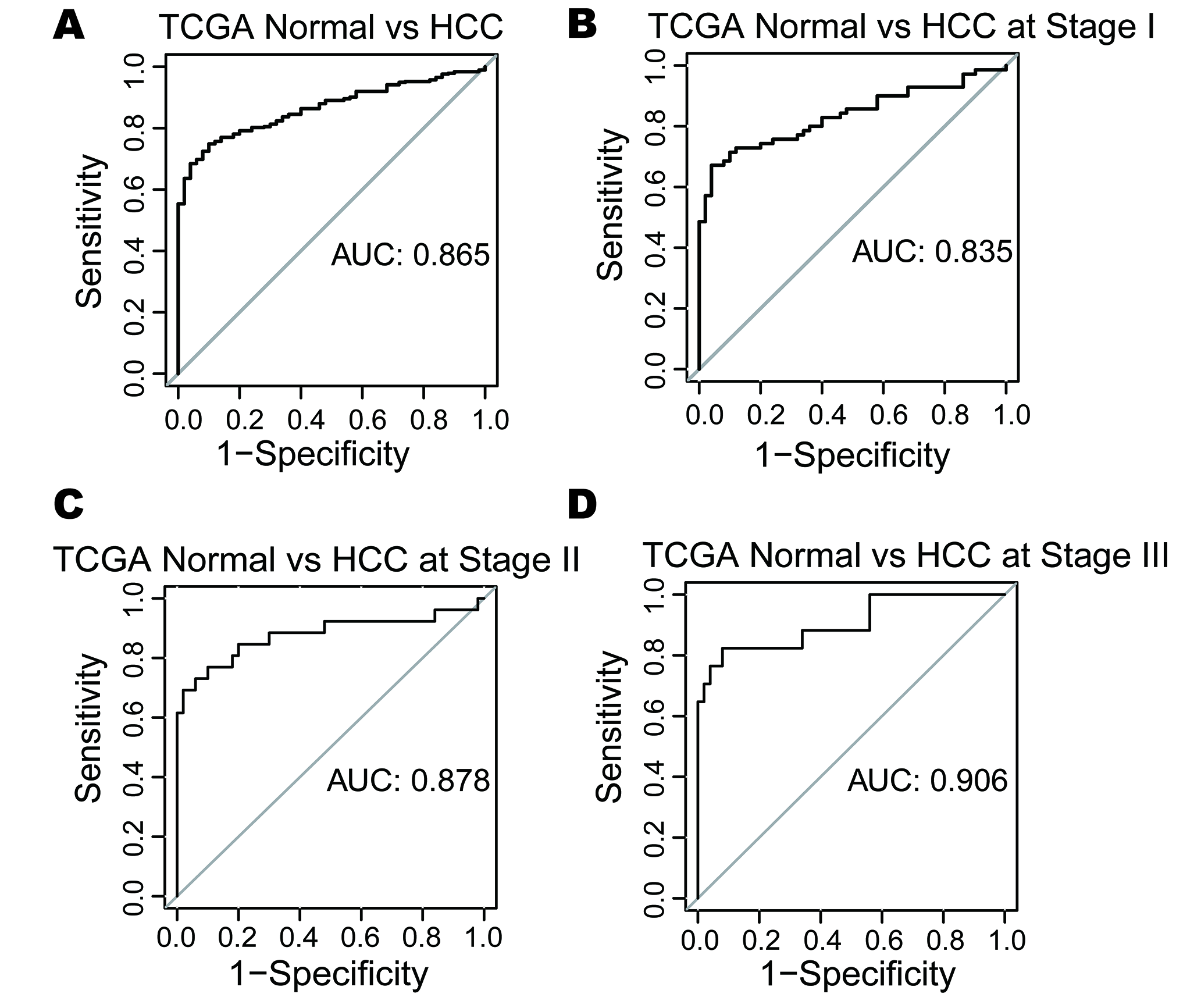

Supplement: Supplementary Figure 3 — GINS4 exhibits a promising capability to discriminate HCC patients from healthy individuals from the TCGA database. ROC curve for (A) all HCC patients, (B) HCC patients at TNM I stage, (C) HCC patients at TNM II stage, and (D) HCC patients at TNM III stage. ROC, the receiver operating characteristic. [file Image_3.tif]

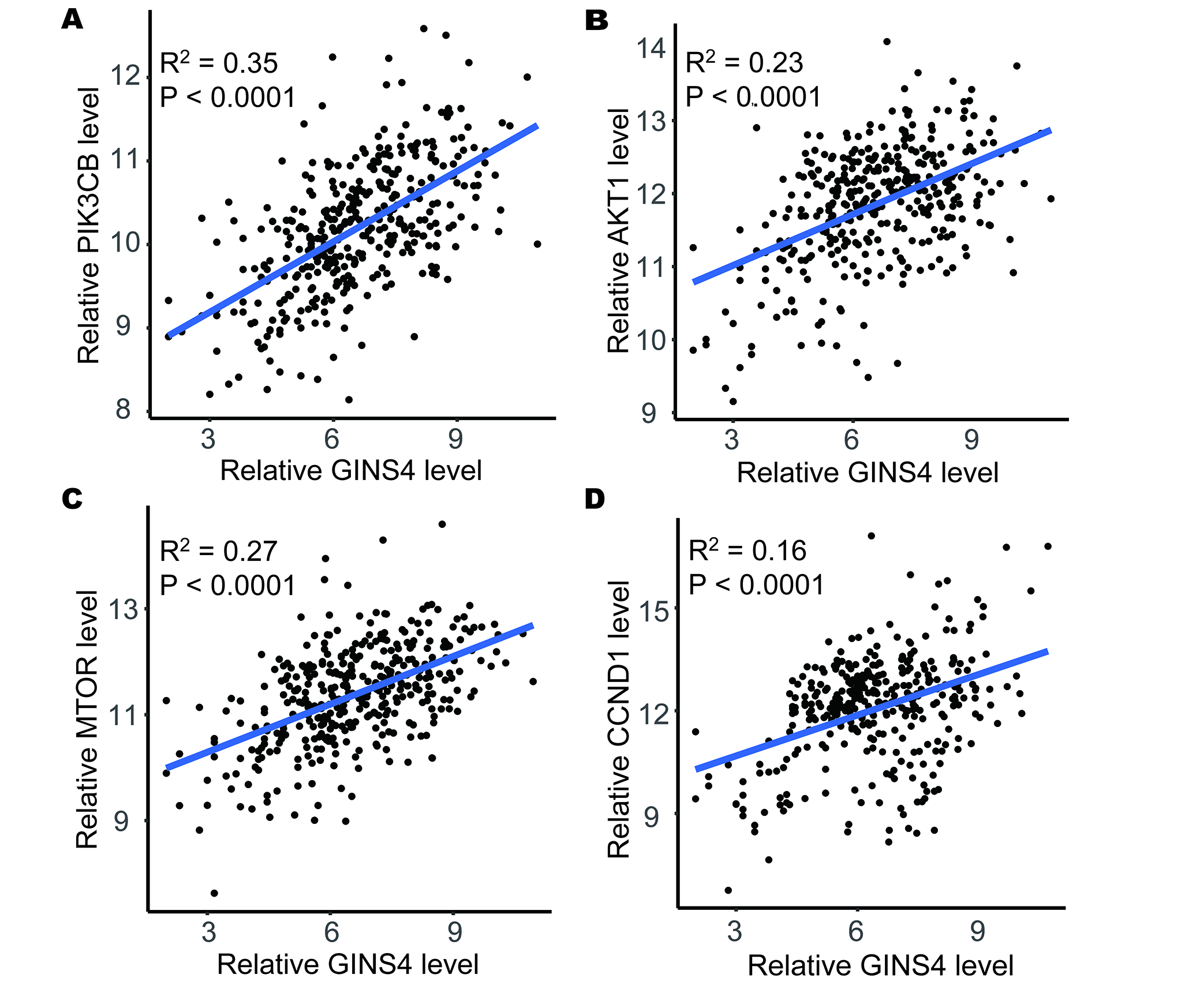

Supplement: Supplementary Figure 4 — GINS4 transcriptome level is positively correlated with genes associated with PI3K/AKT/mTOR pathway in 371 HCC patients from the TCGA database. Pearson correlation analysis revealed the correlation between GINS4 mRNA level and (A) PIK3CB, (B) AKT1, (C) MTOR, and (D) CCND1. [file Image_4.tif]
