# Supplementary material for: Overexpression of GINS4 Is Associated With Tumor Progression and Poor Survival in Hepatocellular Carcinoma
Source: Front Oncol. 2021 Mar 25;11:654185. doi: 10.3389/fonc.2021.654185 (PMC8027117; doi:10.3389/fonc.2021.654185)
Supplement: Supplementary file 5 [file Table_1.docx]

| Methylation site | Chromosome | P value |
| --- | --- | --- |
| cg26367730 | chr8 | 1.57E-15 |
| cg17164093 | chr8 | 2.11E-06 |
| cg07127888 | chr8 | 7.58E-05 |
| cg21487267 | chr8 | 0.00126 |
| cg09196684 | chr8 | 0.126466 |
| cg11196870 | chr8 | 0.169858 |
| cg26554646 | chr8 | 0.206117 |
| cg21517147 | chr8 | 0.845514 |
| cg17835356 | chr8 | 0.878472 |

**Supplementary table 1**: Methylation data of GINS4 in HCC extracted from the Wanderer database.
