# Supplementary material for: Overexpression of GINS4 Is Associated With Tumor Progression and Poor Survival in Hepatocellular Carcinoma
Source: Front Oncol. 2021 Mar 25;11:654185. doi: 10.3389/fonc.2021.654185 (PMC8027117; doi:10.3389/fonc.2021.654185)
Supplement: Supplementary file 6 [file Table_2.docx]

| Gene Symbol | Gene ID | PCC |
| --- | --- | --- |
| [*MCM4*](http://gepia.cancer-pku.cn/detail.php?gene=MCM4) | ENSG00000104738.16 | 0.76 |
| [*RAD51AP1*](http://gepia.cancer-pku.cn/detail.php?gene=RAD51AP1) | ENSG00000111247.14 | 0.76 |
| [*MCM10*](http://gepia.cancer-pku.cn/detail.php?gene=MCM10) | ENSG00000065328.16 | 0.75 |
| [*BRCA1*](http://gepia.cancer-pku.cn/detail.php?gene=BRCA1) | ENSG00000012048.19 | 0.75 |
| [*MCM8*](http://gepia.cancer-pku.cn/detail.php?gene=MCM8) | ENSG00000125885.13 | 0.75 |
| [*NCAPG2*](http://gepia.cancer-pku.cn/detail.php?gene=NCAPG2) | ENSG00000146918.19 | 0.75 |
| [*GINS1*](http://gepia.cancer-pku.cn/detail.php?gene=GINS1) | ENSG00000101003.9 | 0.75 |
| [*DTL*](http://gepia.cancer-pku.cn/detail.php?gene=DTL) | ENSG00000143476.17 | 0.73 |
| [*NUSAP1*](http://gepia.cancer-pku.cn/detail.php?gene=NUSAP1) | ENSG00000137804.12 | 0.73 |
| [*XRCC2*](http://gepia.cancer-pku.cn/detail.php?gene=XRCC2) | ENSG00000196584.2 | 0.72 |
| [*SMC2*](http://gepia.cancer-pku.cn/detail.php?gene=SMC2) | ENSG00000136824.18 | 0.72 |
| [*INCENP*](http://gepia.cancer-pku.cn/detail.php?gene=INCENP) | ENSG00000149503.12 | 0.72 |
| [*ZWINT*](http://gepia.cancer-pku.cn/detail.php?gene=ZWINT) | ENSG00000122952.16 | 0.72 |
| [*MELK*](http://gepia.cancer-pku.cn/detail.php?gene=MELK) | ENSG00000165304.7 | 0.72 |
| [*E2F8*](http://gepia.cancer-pku.cn/detail.php?gene=E2F8) | ENSG00000129173.12 | 0.72 |
| [*STIL*](http://gepia.cancer-pku.cn/detail.php?gene=STIL) | ENSG00000123473.15 | 0.72 |
| [*TOPBP1*](http://gepia.cancer-pku.cn/detail.php?gene=TOPBP1) | ENSG00000163781.12 | 0.72 |
| [*MSH2*](http://gepia.cancer-pku.cn/detail.php?gene=MSH2) | ENSG00000095002.12 | 0.71 |
| [*ESCO2*](http://gepia.cancer-pku.cn/detail.php?gene=ESCO2) | ENSG00000171320.14 | 0.71 |
| [*ARHGAP11A*](http://gepia.cancer-pku.cn/detail.php?gene=ARHGAP11A) | ENSG00000198826.10 | 0.71 |

**Supplementary table 2**: The top 20 most statistically significant genes co-expressed with GINS4 in HCC based on the GEPIA database.
